# Progress in X-Ray Therapeutics

**Published:** 1902-08-30

**Authors:** 


### Progress in X-Ray Therapeutics.

That malignant growths of the skin and of the subcutaneous structures do disappear under treatment with the X-rays there can be no doubt, as numerous reported cases undoubtedly show; but as to whether these disappearances are permanent and the patient really cured of the disease, time alone will show, for the records up to the present are of very recent date. As to how the rays act, according to Leslie Roberts [1] they have a two-fold action—firstly, one analogous to burning, and, secondly, a specific action. "They have a specific action on degenerative epithelium. The growth of hair, nails, and the horny cuticle were rapidly affected, and all these were forms of physiological degenerative epithelium. Rodent ulcer was a purely epithelial disease, and there could be no question that the rays caused a dissolution of the new epithelial cells. The same was true of epithelioma, and possibly to some extent of other forms of cancer. The remedial influence of the rays on lupus was to be attributed to their burning action, and not to any specific influence. No good effect resulted unless the exposure to the rays was followed by at least some symptoms of burning. . . . The X-rays were in the majority of cases inferior to the arc lamp in the treatment of lupus." W. J. Morton [2] suggests that "the analgesic action (of the X-rays) is due to the benumbing of the nerves of sensation. Equally and similarly the remaining nerve supply on the new growth must be benumbed. We have, therefore, a reduction in the force of the total innervation of the new growth. May it not be that this reduction of innervation results in a reduction in the activity of the growth of the part, and thus leads to a retrogression and cessation of its growth?" He further suggests "that the entire protoplasm of the part subjected to the radiation is to a greater or less extent benumbed in its activity," and he calls the process a "paresis or paralysis of the protoplasmic activity." He claims to have produced this "paralysis" in small jelly-fish as they moved about in a salt-water aquarium, by irradiating them with the X-ray.

**Scirrhus.**—Ferguson [1] reports a case in which a recurrent scirrhus growth of the size of a hen's egg disappeared "after twenty applications on as many days," but as there was "some deposit around the axillary vein" at the time of reporting the case, it is evident that the cure was not complete.

**Carcinoma.**—W. J. Morton, New York,[2] amongst other cases, gives four cases of carcinoma treated with the X-ray, in all of which he got great amelioration of symptoms. In one case, recurrent disease had implicated the whole of the left chest over the site of previous operations, and had also invaded the right breast. The effect of treatment was to relieve all the symptoms, and to cause "a marked diminution in size of the new growths, and an entire cessation of pain and suffering." In another case the growths in the breast and on the sternum were much reduced in size, and astonishing relief was given to agonising pain. In a third case, a hard and nodulated tumour of the breast, with an excrescent red growth, the tumour became "softer, more movable and smaller," and the red excrescent area "quite pale and brownish." In the fourth case there was a nodule in the breast the size of a pigeon's egg, hard, and apparently fixed to a rib. The result in this case was "impossible to feel any tumour; none exists, cured, except as to the usual possibility of a recurrence." A. B. Johnson [3] mentions two cases of improvement under treatment: the first was that of a lady who had been twice operated on for cancer, and had a recurrent growth "four inches long and three inches wide, firmly adherent to the ribs, together with several hundred secondary nodules scattered over the skin of the thorax, the upper part of the abdomen and the opposite breast. . . . Without burning this woman, the large growth had been made to practically disappear, the only trace now remaining of the large mass being an area resembling that seen after skin-grafting." The second case was one of carcinoma of the jaw, involving nearly all of the ramus of the jaw. After five-and-twenty applications of the X-rays, "the size of the tumour and of the surrounding soft parts was much less, and the neighbouring lymphatics had diminished in size. The general health had decidedly improved, and from some of the sinuses which still remained open there was a discharge of what appeared to be broken-down carcinoma." T. Bryant of Guy's[4] at a meeting of the South-West London Medical Society gave an account of some cases treated with the X-rays, and amongst them were two cases of recurrent cancer which had derived great benefit from the treatment. He thinks that "we must not yet talk of cancer cures, though we seem justified in saying the X-rays will kill pain and retard growth."

[1] Brit. Med. Journ., Feb. 1, 1902. [2] Med. Rec., Mar. 8, 1902. [3] Med. Rec., Mar. 15, 1902. [4] Brit. Med. Journ., Dec. 7, 1901.

*(To be continued.)*

